# Artificial intelligence prediction of carcinoembryonic antigen structure and interactions relevant for colorectal cancer

**DOI:** 10.1016/j.bbrep.2025.102024

**Published:** 2025-04-21

**Authors:** Ivan Shabo, Erik Nordling, Mirna Abraham-Nordling

**Affiliations:** a Endocrine and Sarcoma Surgery Unit, Department of Molecular Medicine and Surgery, Karolinska Institutet, Stockholm, Sweden; b Swedish Orphan Biovitrum AB, Stockholm, 112 76, Sweden; c Department of Molecular Medicine and Surgery, Karolinska Institute, Stockholm, Sweden

**Keywords:** Carcinoembryonic antigen, Structure prediction, Protein complex, Colorectal cancer

## Abstract

Carcinoembryonic antigen (CEA) is used as a biomarker for colorectal cancer. It is expressed during fetal development but in healthy adult cells the expression is low. Due to its size and the high degree of glycosylation, there are no structures available for mature CEA. By employing novel structure prediction methods, we aim to investigate CEA tertiary structure and interactions.

Alphafold 3 server has increased the accuracy of structure predictions and allows for modelling of glycans in proteins and complexes. Models were created for a monomeric CEA, dimeric CEA and for CEA in complex with the antibody Tusamitamab. The structure of the monomeric glycosylated CEA exhibit two bends, one in the domain interface B1–A2 and one in the domain interface B2-A3. The dimer structure pairs in a parallel manner, with direct contacts in the N and the A2 domains of the two chains. The complex of CEA with Tusamitamab closely resembles the EM structure of the complex that was released after the training of Alphafold 3 was completed.

Overall, the investigations give new angles to investigate for CEA. The predicted bend, primarily in the B2 and A3 domain interface, would allow for dimer formation of CEA from both the same cell as from adjacent cells and could help to explain the outstanding issue on how it can fulfil both tasks. The prediction of the antibody binding to CEA was accurate, the all-atom RMSD was 1.3 Å. This is encouraging for other antibody – protein complexes predictions as the complex structure was not part of the training set for Alphafold 3.

## Highlights

• Prediction of the glycosylated tertiary structure of Carcinoembryonic antigen (CEA).
• Prediction of homodimer of CEA and possible orientation in cell-cell interactions.
• Accurate prediction of CEA and Tusamitamab complex with an all atom RMSD of 1.3 Å.

## Graphical abstract

**Figure:**
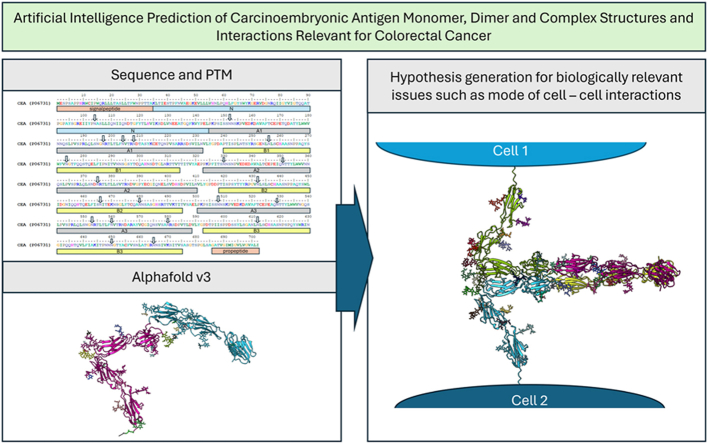

## 1 Introduction

Carcinoembryonic antigen (CEA) is a glycoprotein that participates in cell adhesion and is produced in the gastrointestinal tract during fetal development. In healthy tissues, CEA expression is limited and primarily found in the cells of the digestive system. CEA contains a variable (V)-like domain, named the N domain, followed by six constant C2-like domains named: A1, B1, A2, B2, A3, and B3, that are organized in A-B pairs. CEA is associated with the membrane through a glycosylphosphatidylinositol (GPI) linkage in the C-terminus of the protein [19]. The most common cause of CEA increase in plasma is gastrointestinal malignancy, such as pancreatic and colorectal cancer (CRC) [1,2]. However, plasma CEA may also increase in inflammation, e.g. pancreatitis and Crohn's disease as well as in elderly people and in people who smokes.

Colorectal cancer is the third most common form of cancer worldwide, after lung and breast cancer [3,4]. Emerging evidence highlights that chronic inflammation that promotes a local environment that is favourable for tumor survival and systemic immune response are two key factors in the oncogenesis in colon tissue. One of the pathways that drives the chronic inflammation in CRC are the Toll-like Receptors of the innate immune system, which currently is an active research area [5]. Once the tumor is established it has been shown that the high metabolic demand of cancer cells can cause imbalance in the mitochondria, leading to enhanced proliferation and metastasis [6]. CEA is used to facilitate the detection of CRC or to detect tumor recurrence. It has a certain value for following the treatment effect of palliative care. CEA has too low sensitivity to be used in the normal population or for high-risk groups but can be used in follow-up as a marker for early detection of recurrence [7].

The advent of novel, faster, and more accurate prediction methods for protein structures and protein complexes prompts the investigation of common disease-related proteins. Even though Alphafold has generated the structures of all human proteins and made them available for all in the Alphafold Protein Structure Database [8], they are based on the complete protein sequence, including eventual signal and propeptide that are processed out of the mature protein. It also omits post-translational modifications, such as glycosylations. Even though glycosylation often does not affect the fold of a protein, it might affect the dynamics of protein [9]. Furthermore, in the process of forming a protein complex, the glycosylation will impose steric hindrance and may even shape which areas of the protein surface are available as interactions sites.

The aim of the study was to investigate if novel structure prediction tools may shed light on the structure and function of CEA.

## 2 Material and methods

**Fig. 1:**
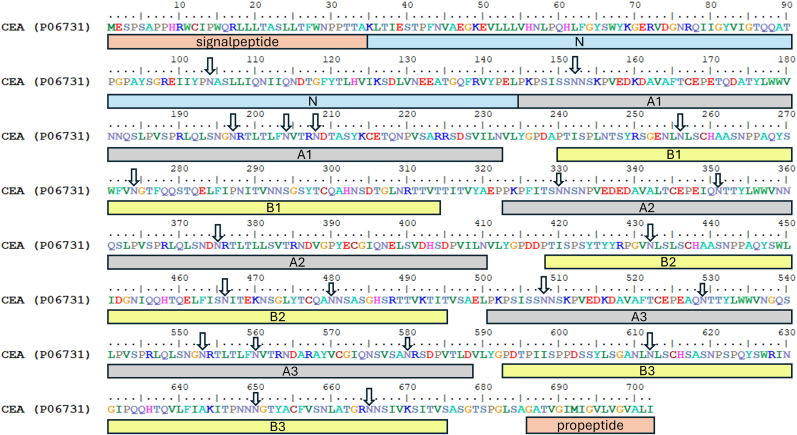
Sequence of CEA (CEAM5_HUMAN, P06731). Annotated with signal and propeptide and domains. The 21 glycosylation sites that are occupied in the predictions are marked with an arrow.

The CEA sequence was collected from Uniprot entry P06731 [10]. After omitting the signal and the pro-peptide from the sequence, residues 35–685 were used as input for the modelling (Fig. 1).

The structure of the mature CEA protein was predicted using the Alphafold 3 server [11]. N-glycans were added based on the occupied glycosylation sites identified by Pont et al. [12]. To each of the following asparagines at position 104, 152, 197, 204, 208, 256, 274, 330, 351, 375, 432, 466, 480, 508, 529, 553, 560, 580, 612, 650 and 665 were a glycan added. Each glycan was modeled as the common core structure of all eukaryotic N-glycans, Manα1-3(Manα1-6) Manβ1-4GlcNAcβ1–4GlcNAcβ1–Asn-X-Ser/Thr [13]. The notation used in Alphafold for the above core structure is NAG(NAG(MAN(MAN)(MAN))).

Dimerization of CEA was modeled by the inclusion of two chains of monomeric CEA in Alphafold 3, described above.

Lastly, modeling was performed with monomeric CEA and the full sequence of the fab of the antibody Tusamitamab, whose ScFv binding to CEA is described in a recent EM study [14].

Result and output files are stored in Nordling [15].

## 3 Results

### 3.1 Monomer structure

**Fig. 2:**
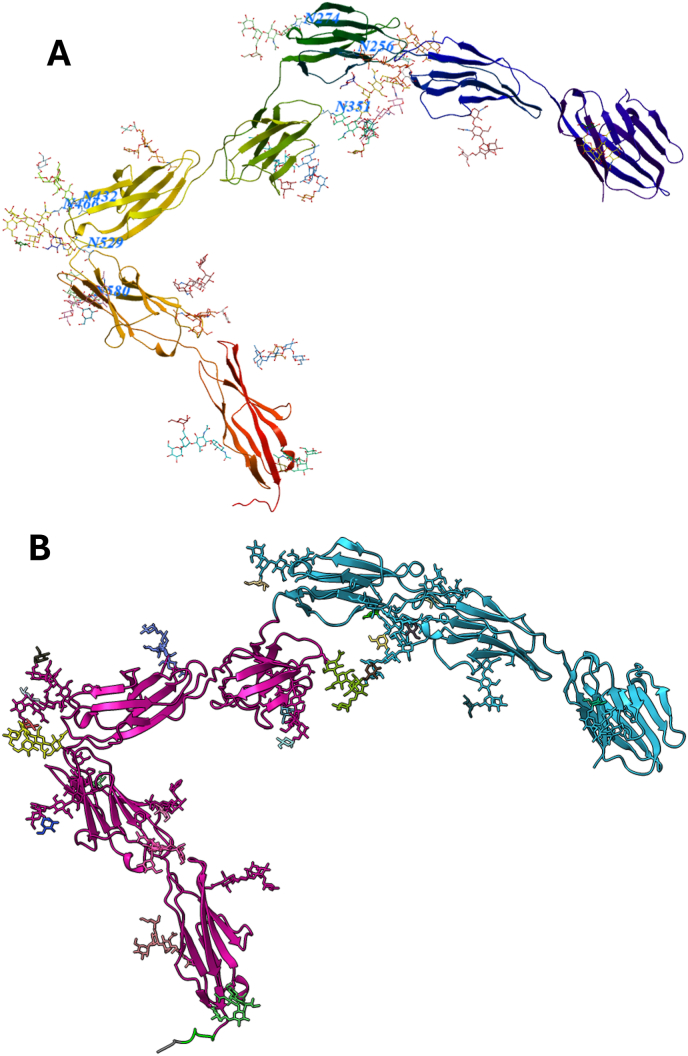
**A.** Predicted structure of CEA monomer with 21 glycans. The N-terminal is collored blue and the c-terminal is colored red. The attachment point to the cellmembrane is located in the c-terminal where an alanine is lapidated and anchors the protein to the cell. Asparagines with glycans close to the bends in the structure are labeled in blue with position and one letter amino acid abbreviation. **B.** Monomer colored according to regions that are predicted with high likelihood of relative position.

The predicted structure for CEA with N-glycans forms an overall elongated structure with the seven Ig-like domains, with two bends formed, the first one between the domain B1 and A2 and the second one between B2 and A3 domain (Fig. 2A). The bend between B1 and A2 domains is caused by a glycan at asparagine 351 that infers steric hindrance between the two domains. This is evident by visual inspection and verified by modeling of the structure without the aforementioned glycan present, where the two domains are positioned more linear (data not shown).

The bend between domains B2 and A3 is not obviously caused by a glycan as removal of all nearby glycans does not abolish the bend; although there are several glycans present in the two domains, they are on the opposite side of the bend. Interestingly, the sequence of the region between these two domains share similarities with each other, and the bend itself is located at a centrally positioned alanine in the linker region between the two domains (Fig. 1). The score of the model is pTM = 0.51 indicating that the overall fold is reasonably correct predicted. The ipTM of 0.5 indicates that some of the relative positions within the model could be erroneous. A plot that depicts which portion of CEA that are predicted with higher certainty is shown in Fig. 2B, where the molecule is divided into two regions that consist of domains 1–3 and 4–7. The relative position of the individual domains within these regions are likely to be predicted accurately. The position of the two regions in relation to each other is however of less certain. This indicates that the more N-terminal bend is predicted with lower probability than the more c-terminal bend, and this is most likely the region that causes the lower ipTM score.

### 3.2 Dimer structure

**Fig. 3:**
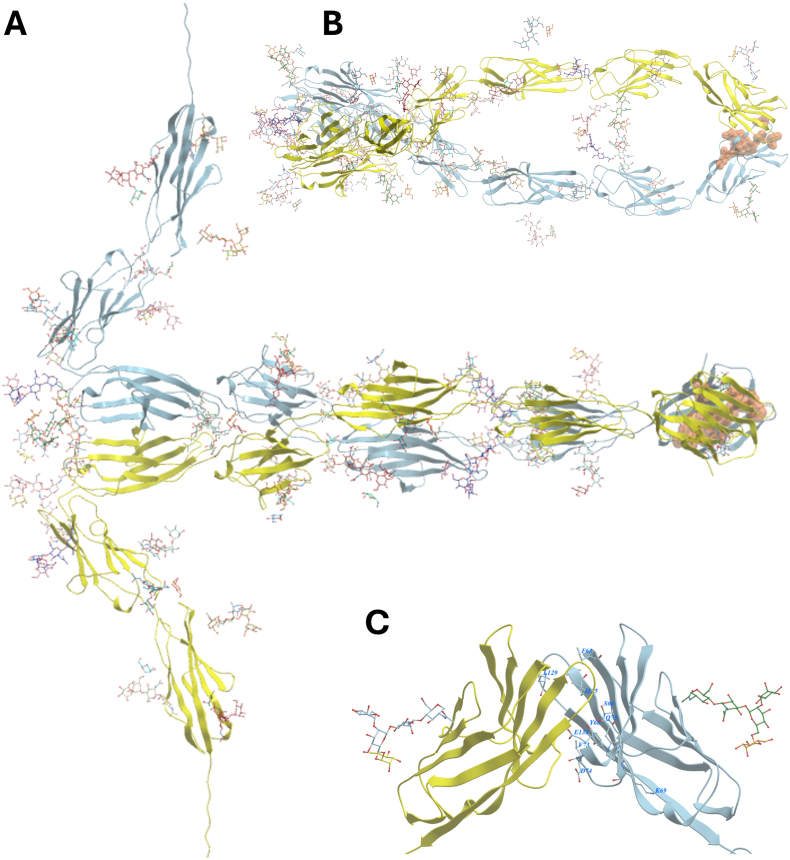
**A.** Predicted CEA dimer, where the first five Ig-like domains form an elongated structure with primary interactions between the n-terminal Ig-like V type domain and the third Ig-like C2 type domain. Residues in the interface regions of the Ig-like V type that have been verified to influence dimer formation are marked in orange. **B.** Dimer rotated 90° into the plane of view to show the interface region more clearly. The central cavity of the dimer that is partly filled with glycans from the first and second of the Ig-like C2 type domains. **C.** Close-up of the n-terminal Ig-like V type domain with residues that are known to influence dimer formation marked with blue labels.

Two plausible models are predicted for the dimer complex. The top-scoring model details interactions between the A2-B2-A3 domains and corresponding domain in the other chain. The C-terminus that is anchored to the plasma membrane with an GPI-anchor, is directed in such that it could imply that it is a dimer protruding from the same cell. However, this conformation of the dimer does not contain any interactions between the variable N domain where point mutations have been shown to disrupt dimer-formation [[16], [17]]. The second-best scoring dimer model includes direct interactions between the N domains of the two chains (Fig. 3A). The glycans of the A1 and B1domains are pointing into a vacant area in between the protein chains, causing the dimer to form an extended version of a Fc domain of an antibody that is ended by direct interactions of the A2 domain, which in turn forms extensive contacts through the edge of one of the beta-sheets of the Ig-fold (Table 1, Fig. 3B). The mutated residues that have been shown to disrupt dimer formation Korotkova et al., 2008, [17] are positioned in the interface region of the two N domains (Table 1, Fig. 3C). The membrane attachment points are directed toward opposite sides, indicating that this dimer formation possibility is compatible with accommodate cell-cell interactions. The third-best model forms a similar structure as the second-scoring model, but the N domains are rotated 180° and form a dimer with an interface that do not contain the mutationally verified residues that influence dimer formation.

**Table 1:** Detailed list of interactions between domains in the dimer structure. Residues that are reported to interrupt dimer formation are marked with bold text and an asterisk.

Chain 1: Domain	Residues	Type of interaction	Residues	Chain 2: Domain
N	**Phe63∗** sidechain	hydrophobic	**Phe63∗** sidechain	N
	**Phe63∗** sidechain	hydrophobic	Leu129 sidechain	
	Gly64 backbone Ca	hydrophobic	**Ile125∗** sidechain	
	**Ser66∗** sidechain	hydrogen bond	Leu129 backbone O	
	**Tyr68∗** sidechain	hydrogen bond	Asn131 sidechain	
	Glu71 sidechain	salt bridge	Arg72 sidechain	
	Arg72 sidechain	salt bridge	Glu71 sidechain	
	**Val73∗** sidechain	hydrophobic	**Val73∗** sidechain	
	**Gln78∗** sidechain	hydrogen bond	Leu129 backbone O	
	Val83 sidechain	hydrophobic	Leu129 sidechain	
	Thr90 sidechain	hydrophobic	Val130 sidechain	
	His123 sidechain	hydrogen bond	His123 sidechain	
	**Ile125∗** sidechain	hydrophobic	Leu129 sidechain	
	Leu129 sidechain	hydrophobic	**Phe63∗** sidechain	
	**Glu133∗** sidechain	hydrogen bond	Gly75 backbone N	
A1	Asn152 Glycan	hydrophilic	Asn152 Glycan	A1
	Asn204 Glycan	hydrophilic	Asn204 Glycan	
B1		no contacts		B1
A2	Phe326 sidechain	hydrophobic	Asn330 sidechain	A2
	Ile327 backbone N	hydrogen bond	Asn330 backbone O	
	Ile327 backbone O	hydrogen bond	Asn330 backbone N	
	Ser329 backbone N	hydrogen bond	Ser329 backbone O	
	Ser329 backbone O	hydrogen bond	Ser329 backbone N	
	Asn330 sidechan	hydrophobic	Phe326 sidechain	
B2		no contacts		B2
A3		no contacts		A3
B3		no contacts		B3

### 3.3 CEA and FAb complex structure

**Fig. 4:**
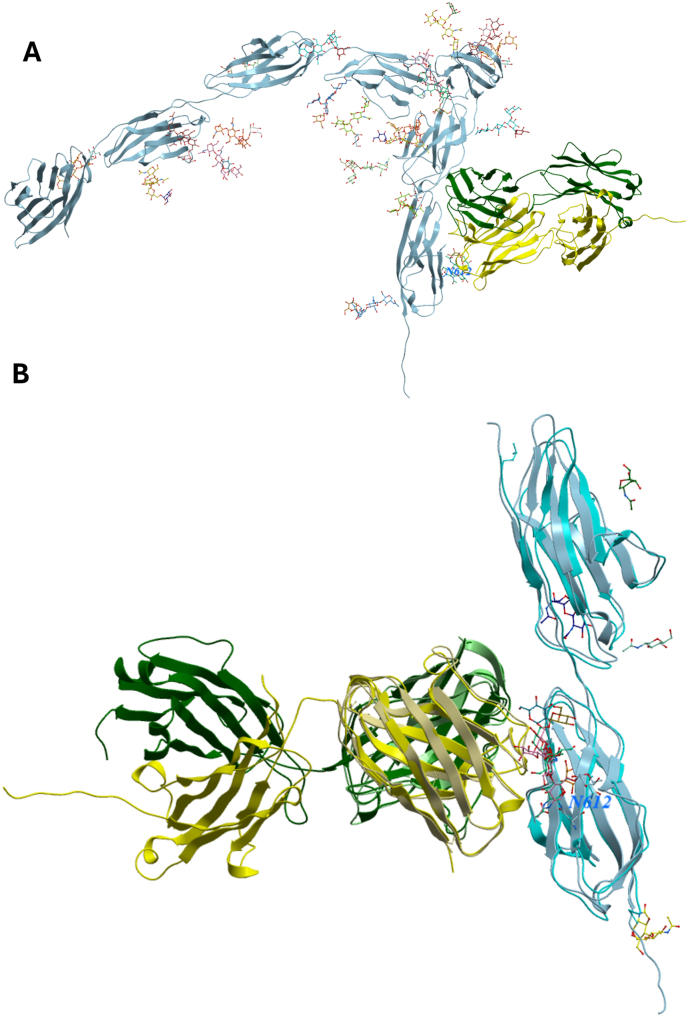
**A.** Structure of CEA, colored in light-blue, bound to the FAb of Tusimatimab, colored in green and yellow. **B.** Comparison of EM structure of complex of ScFv of Tusimatimab (colored in khaki and light-green) and CEA Ig-like C2 type domain 5 and 6 (colored in cyan) and the corresponding portion of the complex of the FAb and CEA, the predicted structure is colored as in A.

The complex of the full-length CEA with glycans and the FAb of Tusamitamab captures the information from the EM structure, including both the protein interactions and the interaction with the Glycan at Asn612. (Fig. 4A). Interestingly, the overall model quality score pTM is higher than for the monomer itself at 0.52, while the score that relates to domain relations, ipTM are the same, 0.5. Overlay with the EM structure in 8bw0 show that the all-atom RMSD is 1.3 Å for the fragment and the two domains of CEA included in the EM structure (Fig. 4B). This structure was released on 2024-01-24 which is after the cutoff date of 2021-09-30 for the training set of Alphafold 3 [14].

## 4 Discussion

The structure of the monomer with added glycans depart from the structure that is predicted without glycans present and the one of the sequence of the immature chain, including signal and propeptide that is deposited in the Alphafold database (https://alphafold.ebi.ac.uk/entry/P06731). The current prediction has a bend in the structure in the linker region between B1 and A2 domains and that creates a sharp angle between B2 and A3 domains. The structure of the first bend is predicted with low certainty, it is influenced strongly by the glycan at position 351, as the removal of that glycan predicts a more elongated orientation at this linker region. The other region is less influenced by local interactions with glycans and the driver of the formation of the sharp bend is to hide a hydrophobic patch of residues, including Tyr424, Tyr426, Leu500, Pro501, Pro525, Ala527. These two regions are predicted with lower certainty in the unglycosylated models these regions might be flexible and adopt several positions in relation to each other. Another possible explanation is that the algorithm is skewed towards trying to hide these hydrophobic regions when more hydrophilic groups are present in the simulation.

Previous modeling based on EM data suggests a more elongated form of length 27–33 nm and a cylindrical shape of dimension 8 nm × 1 nm. In these experiments, dimers might form and what is actually measured could be the homodimer of CEA. Upon inspection of the dimer prediction, the first five domains of CEA form an elongated structure that is 8.5 nm wide and 3 nm high, which is in agreement with the published EM data. However, the length of the portion is only 21 nm. The two c-terminal domains are approximately 8 nm long and angled out of the plane. But, if these were more in line with the other domains, the distance would match the EM data. The bend might be a flexible hinge in CEA allowing the CEA dimer formation to accommodate from both the same cell and two separate cells [18]. In this manner, the dimer formation that is observed for the N-terminal domain can be satisfied by both trans and cis dimer formation of CEA [16].

The modeling of the complex with the Fab of the Tusimatimab antibody bound to CEA monomer creates a similar overall structure for CEA that is consistent with the previously reported structure of the ScFv CEA complex. The RMSD of all atoms 1.3 Å. This is a remarkable accomplishment of the algorithm, as the structure of the ScFv-CEA complex was released 18 months after the training set for Alphafold 3 was compiled. Even the influence of the glycan at position 612 is predicted.

## 5 Conclusion

Overall, the predictions present a possible interaction model that allows for dimer formation of CEA in both cis and trans orientation regarding position on cells, i.e. CEA located on the same cell or on different cells. The bent conformation will allow for the binding of CEA molecules from different cells as the interaction area is presented facing upwards from the cell surface. As the regions between the Ig-like domain are flexible, we assume that the same orientation driven by the interaction of the N-terminal Ig-like V-type domain can achieve *cis*-dimerization. This allows to the design of directed experiments to verify the proposed complex.

## CRediT authorship contribution statement

**Ivan Shabo:** Writing – review & editing, Visualization, Investigation. **Erik Nordling:** Writing – review & editing, Visualization, Validation, Methodology. **Mirna Abraham-Nordling:** Writing – review & editing, Writing – original draft, Project administration, Methodology, Conceptualization.

## Funding information

No funding was received for the current study.

## Declaration of competing interest

The authors declare the following financial interests/personal relationships which may be considered as potential competing interests: Co-author Erik Nordling is currently employed by Swedish Orphan Biovitrum AB. The company has no financial or scientific interest in the published work. If there are other authors, they declare that they have no known competing financial interests or personal relationships that could have appeared to influence the work reported in this paper.
